# Transcutaneous CO_2_ Monitoring in Extremely Low Birth Weight Premature Infants

**DOI:** 10.3390/jcm12175757

**Published:** 2023-09-04

**Authors:** Liron Borenstein-Levin, Noa Avishay, Orit Soffer, Shmuel Arnon, Arieh Riskin, Gil Dinur, Karen Lavie-Nevo, Ayala Gover, Amir Kugelman, Ori Hochwald

**Affiliations:** 1 Department of Neonatology, Rambam Health Care Campus, Haifa 3109601, Israel; oritsof@gmail.com (O.S.); gil.dinur@gmail.com (G.D.); amirkug@gmail.com (A.K.); o_hochwald@rambam.health.gov.il (O.H.); 2 Rappaport Faculty of Medicine, Technion-Israel Institute of Technology, Haifa 3200003, Israel; noa.avishai@gmail.com (N.A.); arik.riskin@gmail.com (A.R.); klavie@gmail.com (K.L.-N.); ayalagover@gmail.com (A.G.); 3 Department of Neonatology, Meir Medical Center, Kfar-Saba 4428164b, Israel; shmuelar@clalit.org.il; 4 Faculty of Medicine, Tel Aviv University, Tel Aviv 69978, Israel; 5 Department of Neonatology, Bnai Zion Medical Center, Haifa 32000, Israel; 6 Department of Neonatology, Carmel Medical Center, Haifa 3436212, Israel

**Keywords:** non-invasive CO_2_ monitoring, premature infant, transcutaneous CO_2_ monitoring

## Abstract

Extremely low birth weight (ELBW) premature infants are particularly susceptible to hypocarbia and hypercarbia, which are associated with brain and lung morbidities. Transcutaneous CO_2_ (TcCO_2_) monitoring allows for continuous non-invasive CO_2_ monitoring during invasive and non-invasive ventilation and is becoming more popular in the NICU. We aimed to evaluate the correlation and agreement between CO_2_ levels measured by a TcCO_2_ monitor and blood gas CO_2_ (bgCO_2_) among ELBW infants. This was a prospective observational multicenter study. All infants < 1000 g admitted to the participating NICUs during the study period were monitored by a TcCO_2_ monitor, if available. For each bgCO_2_ measured, a simultaneous TcCO_2_ measurement was documented. In total, 1828 pairs of TcCO_2_–bgCO_2_ values of 94 infants were collected, with a median (IQR) gestational age of 26.4 (26.0, 28.3) weeks and birth weight of 800 (702, 900) g. A moderate correlation (Pearson: r = 0.64) and good agreement (bias (95% limits of agreement)):(2.9 [−11.8, 17.6] mmHg) were found between the TcCO_2_ and bgCO_2_ values in the 25–70 mmHg TcCO_2_ range. The correlation between the TcCO_2_ and bgCO_2_ trends was moderate. CO_2_ measurements by TcCO_2_ are in good agreement (bias < 5 mmHg) with bgCO_2_ among premature infants < 1000 g during the first week of life, regardless of day of life, ventilation mode (invasive/non-invasive), and sampling method (arterial/capillary/venous). However, wide limits of agreement and moderate correlation dictate the use of TcCO_2_ as a complementary tool to blood gas sampling, to assess CO_2_ levels and trends in individual patients.

## 1. Introduction

Extremely premature infants are susceptible to hyper- or hypocapnia and rapid fluctuations in PaCO_2_, especially during the first week of life [1]. While monitoring PaCO_2_ in a blood sample is the “gold standard”, it only allows for interval monitoring and not continuous monitoring. Thus, periods of abnormally high or low PaCO_2_ may be missed, and corrective ventilation measurements may be delayed. 

Two methods that allow for non-invasive, continuous CO_2_ monitoring in the NICU are End-tidal CO_2_ (EtCO_2_) monitoring and Transcutaneous CO_2_ (TcCO_2_) monitoring. In EtCO_2_ monitoring, the capnograph sensor is connected to the endotracheal tube and allows for mainstream or side-stream measurements of EtCO_2_ [2]. EtCO_2_ monitoring was found to have a good correlation with bgCO_2_ among ventilated term and preterm infants [3,4], though the agreement was only moderate during the first day of life [5], and was negatively influenced by the severity of lung disease [4,6,7]. Among infants receiving mechanical ventilation in the NICU, the use of continuous EtCO_2_ monitoring was found to improve the control of CO_2_ levels within a safe range. In a subgroup analysis of extremely low birth weight premature infants (ELBW), the prevalence of intraventricular hemorrhage and periventricular leukomalacia was lower in the EtCO_2_-monitored group; however, this group was too small to draw firm conclusions [8]. The main clinical limitation of EtCO_2_ monitoring in the neonatal intensive care unit (NICU) is that it cannot be used in infants supported by high-frequency oscillatory ventilation (HFOV) or non-invasive ventilation, which are ventilation modes that are commonly used in this population [2].

TcCO_2_ is based on the ability of CO_2_ to diffuse through body tissues and skin and be detected by a sensor on the surface of the skin. By warming the sensor, local hyperemia is induced, which increases the supply of arterial blood to the dermal capillary bed below the sensor [9]. TcCO_2_ monitors are currently widely used in the NICU [10,11]. Historically, neonatal studies have shown that TcCO_2_ correlates better with PaCO_2_ compared to EtCO_2_ [12,13,14], though more recent studies revealed inconclusive results [5,15,16,17].

Given the importance of avoiding extreme CO_2_ values and fluctuations during the first week of life among ELBW premature infants, the growing popularity of TcCO_2_ monitoring in the NICU, and the inconclusive data regarding their accuracy in this population, we conducted this study. Our aim was to evaluate the correlation and agreement between CO_2_ levels measured by the TcCO_2_ monitor and blood gas CO_2_ (bgCO_2_) among ELBW infants during their first days of life. We hypothesized that TcCO_2_ monitoring will be in good correlation and agreement with bgCO_2_ measurements as well as CO_2_ trends

## 2. Materials and Methods

These data were part of a prospective, observational, multicenter study studying the impact of TcCO_2_ monitoring on neurologic and respiratory complications among ELBW infants (under submission). This study was approved by the research ethics board of all centers participating in the study. Written informed consent was obtained from the parents of all infants prior to study entry.

### 2.1. Study Population

All premature infants < 1000 g admitted to the participating NICUs during the study period and needing respiratory support during the first day of life were monitored by TcCO_2_ monitor (Sentec AG, Therwil, Switzerland), if available, during the first week of life or longer as clinically indicated. Respiratory support included invasive support (Conventional mechanical ventilation (CMV) and HFOV) and non-invasive support including nasal intermittent positive pressure ventilation (NIPPV), continuous positive airway pressure (CPAP), and heated humidified high flow nasal cannula (HHHNC).

Infants with severe congenital malformation, birth asphyxia, known intraventricular hemorrhage stage III–IV in the first 24 h of life, or if active treatment was not initiated were excluded from the study. 

### 2.2. Study Design

TcCO_2_ monitoring was started during the first 12 h of life. Probe placement was in predefined areas as per manufacturer instructions. The sensor temperature was set to 41 °C in accordance with the manufacturer’s instructions [18]. Calibration of the TcCO_2_ was automatically performed every 4 h and following any reposition of the probe. Sensor membranes were changed every 28 days or sooner in case of any visible damage or repeated calibration errors. Skin fixation adhesives and contact gel were used in accordance with manufacturer guidelines.

Blood samples were taken at the discretion of the bedside care team, following meticulous placement of the probe and allowing for an adequate time period to achieve equilibrium. For each blood sample drawn for blood gas monitoring, a simultaneous TcCO_2_ measure was recorded, as well as other clinical and respiratory support data. 

### 2.3. Statistical Analysis

Data are presented as mean ± standard deviation (SD) for normally distributed variables, or median with interquartile range (IQR) for variables with non-parametric distribution. The correlation between TcCO_2_ and bgCO_2_ was measured using Pearson correlation. To determine the agreement between the two CO_2_ measuring methods, a Bland–Altman analysis was performed on all matched TcCO_2_–bgCO_2_ samples, correcting for multiple measurements per patient [19]. Data are presented as bias (mean difference) and 95% limits of agreement (LoA) (i.e., 1.96 times the SD of the bias). The correlation of measurement trends was assessed for all consecutive pairs of TcCO_2_ and bgCO_2_ using Pearson correlation. 

Logistic regression analysis was used to examine the relationship between different variables examined and the likelihood that the TcCO_2_–bgCO_2_ difference will be <|5|, which we consider clinically acceptable [3]. We incorporated into the model risk factors with *p* value < 0.05.

Statistical analyses were performed with SPSS version 25 (IBM SPSS, Chicago, IL, USA). Bland–Altman plot according to multiple measurements per subject was performed by MedCalc^®^ Statistical Software version 20.218 (MedCalc Software Ltd., Ostend, Belgium).

## 3. Results

The study was conducted between March 2018 and September 2021 in the NICU’s in Rambam, Bnai Zion, Meir, and Carmel medical centers. A total of 1828 pairs of TcCO_2_ and bgCO_2_ of 94 ELBW premature infants were collected, with a median (IQR) GA of 26.4 (26.0, 28.3) weeks and birth weight of 800 (702, 900) g. Demographic data are presented in Table 1.

**Figure 1:**
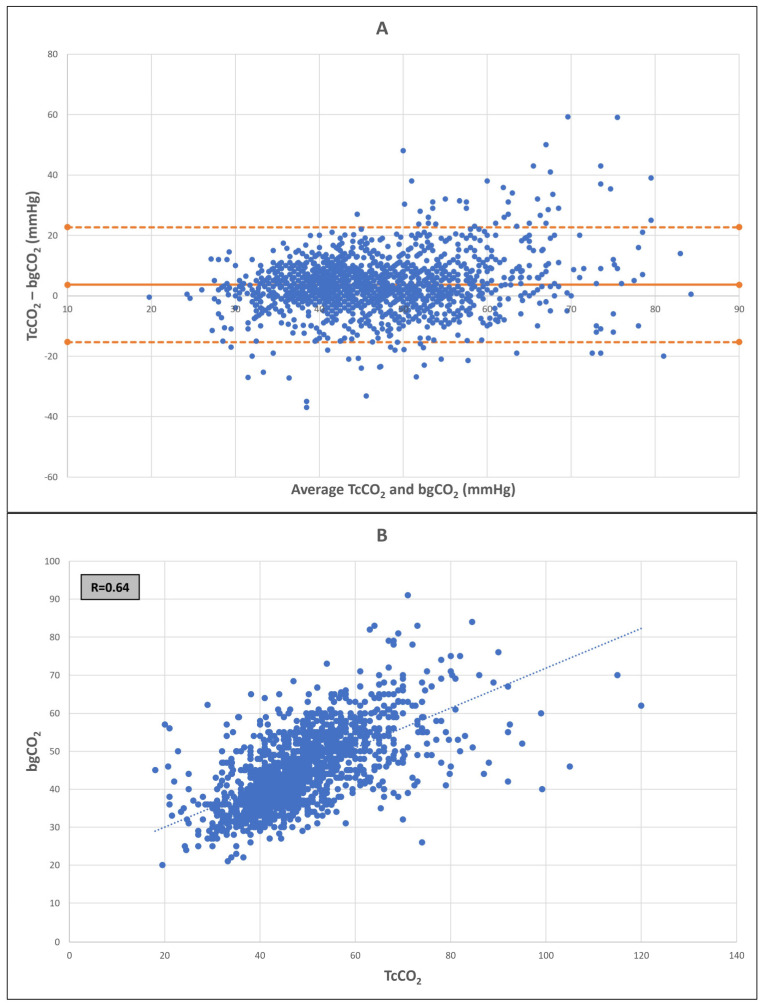
(**A**) Bland–Altman plot of the differences between TcCO_2_ and bgCO_2_. Orange lines represent the bias (solid line) and 2SD (dotted lines). (**B**) Pearson correlation between TcCO_2_ and bgCO_2_. bgCO_2_—blood gas CO_2_; TcCO_2_—transcutaneous CO_2_.

The Bland–Altman analysis showed a mean bias of 3.6 mmHg with a 95% confidence LoA from −14.3 to +21.4 mmHg (Figure 1A). Pearson’s correlation coefficient between TcCO_2_ and bgCO_2_ was r = 0.64 (Figure 1B). The corrected Bland–Altman analysis according to multiple measurements per subject showed similar results (mean bias of 3.6 mmHg with a 95% confidence LoA from −14.1 to +21.2 mmHg).

Similarly, moderate correlation and good agreement were demonstrated in TcCO_2_ values ranges of 30–60 mmHg and 25–70 mmHg (the ranges that are most frequently seen at the bedside) (Table 2). For TcCO_2_ below 25 and above 70 mmHg the correlation was poor (r = −0.41 and 0.14, respectively) as was the agreement (bias (LoA) −16.3 [−40.0, 7.4] and 20.1 [−9, 49.1] mmHg, respectively). However, the number of samples at these extremes was small.

The CO_2_ range for TcCO_2_ was 18–120 mmHg and for bgCO_2_ was 20–91 mmHg.

Ninety-six percent of the samples were taken during the first week of life. Samples taken during the first 3 days of life had a stronger correlation and lower bias but still a wide LoA. Similar results are seen for venous samples as compared to arterial or capillary. Samples taken during non-invasive ventilation had a similar correlation and agreement as samples taken during the different invasive ventilation modes (HFOV and CMV) (Table 2).

In 950 out of 1724 of the samples (55%), the TcCO_2_ reading was within the ±5 mmHg range as compared to bgCO_2_. A total of 491/1724 (29%) were within the 6–10 absolute difference range, and in 283/1724 samples (16%), the difference was >10.

Multivariable logistic regression showed that sampling during the first 3 days of life and venous sampling significantly increase the likelihood that the TcCO_2_–bgCO_2_ difference will be less than or equal to five (95% CI for first 3 days of life—1.52 [1.24–1.87], *p* < 0.001, and for venous sampling—1.87 [1.16–3.01], *p* = 0.01), while HFOV increases the likelihood of absolute difference greater than five (95% CI 0.78 [0.59–0.97], *p* = 0.037).

**Figure 2:**
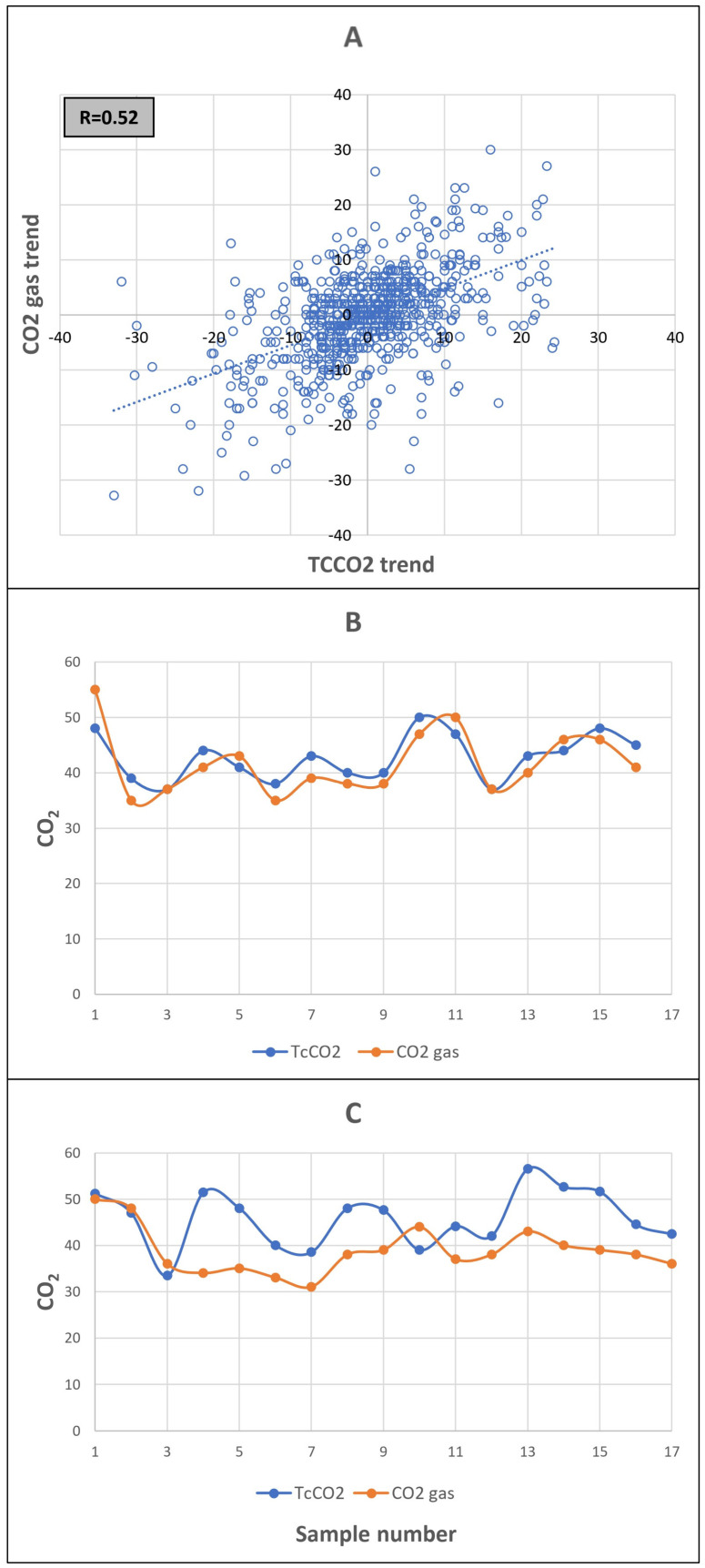
Comparison of the trending of TcCO_2_ and bgCO_2_: (**A**) Scatter plot of the change in the measured value between 2 consecutive measurements in bgCO_2_ vs. TcCO_2_ during the first 3 days of life (*n* = 657). (**B**,**C**) Examples of the trends in individual infants. Example B demonstrates a good agreement and trending between TcCO_2_ and bgCO_2_ measurements, while in example C, the agreement as well as trending is changing.

To evaluate the trending accuracy of TcCO_2_, we studied samples taken during the first 3 days of life. We chose this time period because, in the first days of life, blood gas sampling is usually more frequent and therefore we avoided, as much as possible, studying samples taken more than 12 h apart. A moderate correlation was found between the trending of each two successive measurements of TcCO_2_ vs. bgCO_2_- r = 0.52 (Figure 2A). However, studying individual infants, we observed a good correlation in CO_2_ trends in some infants while a poor trend in others (Figure 2B,C).

We did not observe any burns or skin breakdowns among the participating infants.

## 4. Discussion

In this large, prospective, multicenter study, we found a moderate correlation between transcutaneously measured CO_2_ values and blood gas CO_2_, among ELBW premature infants during their first week of life; a period when they are especially vulnerable to the harms of both hypocarbia and hypercarbia. The agreement between the two measuring methods was good; however, a wide limit of agreement exists.

The accuracy of TcCO_2_ monitoring among premature infants was previously studied in the NICU in various clinical situations. Mukhopadhyay et al. [20] analyzed 1338 paired samples of TcCO_2_ and bgCO_2_, of mostly premature infants (mean ± SD GA 28.6 ± 4.3), in two different time periods, and found a bias ± SD of 5.2 ± 8.6 mmHg. Aliwalas et al. [5] studied 81 pairs of samples of intubated preterm infants ≤ 28 weeks gestation with RDS at 4, 12, and 24 h of age and showed bias ± SD of 2.2 ± 2.3, 4.4 ± 1.2, and 2.6 ± 1.8 mmHg, respectively. Van Weteringen reported a bias of 4.7 mmHg (95% LoA −7.8 to 17.1 mmHg) in 216 paired samples of premature infants (median (IQR) GA 26.4 [25.3–27.5]) with a similar agreement in subgroup analysis based on birth weight (below or above 1000 g), week of life (during or after the first week of life), and sepsis status (no sepsis, suspected and proven sepsis) [21]. A good correlation and agreement were also demonstrated when using a reduced temperature probe [18,22]. A poor correlation was found by Janaillac et al. [23]; however, these results should be addressed with caution as the average time lag between the pairs of samples was 4 min.

In our study, we focused on a homogenous group of ELBW premature infants during their first week of life, when they are most vulnerable to both hypocarbia and hypercarbia [24]. Studying 1828 paired samples, we found a bias of 3.6 mmHg, which is considered acceptable (<5 mmHg), with LoA from −14.3 to +21.4 mmHg. These results are comparable to previous studies and highlight the advantages of this CO_2_ monitoring method—it is reliable, and it allows the continuous non-invasive monitoring of CO_2_ in ELBW infants supported by all modes of invasive or non-invasive ventilation. Our study also demonstrates the disadvantage of this method, which is the wide LoA, also reported by others who have studied TcCO_2_ monitoring [18,20,21]. A wide LoA was found also for EtCO_2_ monitoring [3,4,6,7]. This emphasizes the importance of combining these methods with blood gas sampling, as these two non-invasive methods, TcCO_2_ and EtCO_2_, cannot be used as independent indicators of CO_2_ levels.

Studying the impact of hemodynamic stability including blood pressure, oxygenation, arterial pH, and medications on TcCO_2_, Bhat et al. found that the major factors affecting the TcCO_2_ to bgCO_2_ agreement were hypoxia and acidosis [25]. We were able to demonstrate similar agreement during the first days of life when the hemodynamic stability and oxygenation of ELBW infants are a concern, and it is reassuring that TcCO_2_ is indeed a reliable method for CO_2_ monitoring in this population.

In our study, we chose to focus on measurements between 25 and 70 mmHg as measurements above 70 mmHg and below 25 mmHg were found to have poor correlation and agreement. Poor correlation in the hypercarbia range was also demonstrated by Uslu et al. [26] and is suggested to result from impaired capillary blood flow and gas diffusion to the skin when the pH decreases. Interestingly, in the hypocapnia range, the bias was inverted, showing TcCO_2_ measurements lower than bgCO_2_ measurements. Low TcCO_2_ readings that fall below the bgCO_2_ value may indicate a technical problem as TcCO_2_ values are generally higher than PaCO_2_ values due to a local increase in CO_2_ by the elevated temperature and by CO_2_ production of epidermal cells [9]. This is also demonstrated by a mean bias > 0 mmHg. It is possible that the small number of measurements in the extreme values of CO_2_ is the reason for the poor correlation and agreement in these ranges. We suggest, in any case, to exercise caution when interpreting TcCO_2_ measurements in the extreme ranges.

Other studies found that the sampling method or mode of ventilation could affect the accuracy of TcCO_2_ measurements. For example, Mukhopadhyay et al. found that HFOV support significantly increases the odds of increased bias [20], and others found that tcCO_2_ was more accurate for capillary blood samples than for arterial blood samples [16,20,27]. In our study, 84% of the samples were within an absolute range of ±10 mmHg. We found a slight improvement in correlation and reduced bias in venous samples, and samples taken during the first 3 days of life. No statistical differences were found in samples collected while infants were on CMV or HFOV (Table 2). In multivariate analysis, venous sampling was associated with bias < 5 mmHg and HFOV with bias > 5 mmHg. However, these small differences are purely statistical and have no clinical significance.

As expected, TcCO_2_ was also accurate during non-invasive ventilation. These results are reassuring as one of the main advantages of monitoring CO_2_ transcutaneously is the ability to use it during non-invasive ventilation and during HFOV, which is technically challenging with other modes of non-invasive CO_2_ monitoring [2].

TcCO_2_ monitoring is suggested to be used as a complementary tool to blood gas sampling to allow trending of CO_2_ levels. TcCO_2_ trends have been successfully used to identify optimal lung volume during HFOV in neonates [28] and are proposed to allow early diagnosis of pneumothorax [29]. During the first 3 days of life, we found a moderate correlation between the TcCO_2_ trends and bgCO_2_ trends. We noticed excellent trending in some infants while poor trending in others. This observation reinforces the need to ascertain the trending in each individual patient, and a high index of suspicion whenever the TcCO_2_ measurement does not fit the clinical scenario.

The main limitation of our study is that the samples were taken according to clinical need and not at a predetermined interval, which could have better delineated the trend-monitoring ability of this monitoring method. Another limitation is that the number of measurements per infant varies, but this was corrected by Bland–Altman analysis according to multiple measurements per subject. Furthermore, we did not record the sensor location and time from the last calibration. This prevented us from further studying the sensor location effect on the accuracy of the measurements as well as assessing the technical challenges associated with sensor positioning in the high-humidity environment required for ELBW during the first weeks of life. However, sensor location and calibration were performed as per the manufacturer’s instructions; therefore, it represents the standard practice. The large number of samples most probably compensates for any false samples, if any. Due to the small number of infants with active sepsis or ionotropic support, we could not perform a multifactorial analysis to isolate parameters that could affect perfusion, as reported by others [30]. The advantages of our study are the large number of samples, the prospective nature of the study, and the focus on ELBW infants during their first week of life; the most vulnerable population during the most critical time period for CO_2_ fluctuations.

## 5. Conclusions

CO_2_ measurements by TcCO_2_ have a moderate correlation with bgCO_2_ among premature infants < 1000 g during the first week of life. While agreement between the TcCO_2_ and bgCO_2_ measurements is good, the wide LoA, as well as the moderate correlation of trends, dictate the use of this continuous non-invasive method as a complementary tool along with blood gas sampling to assess CO_2_ levels and trending.

## Figures and Tables

**Table 1:** Demographics.

Premature Neonates *n* = 94	
Gestational Age, weeks	26.4 (26.0, 28.3)
Birth weight, g	800 (702, 900)
Small for gestational age	8 (8)
Prenatal steroids	65 (69)
Preeclampsia	25 (27)
Multiple births	26 (28)
Male gender	40 (43)
Delivery mode—Cesarean section	72 (77)
Apgar 5’	8 (6, 9)
Intubation at delivery room	41 (44)
Umbilical cord pH	7.27 (7.19, 7.33)
RDS requiring surfactant treatment	56 (60)
Ionotropic support during first week	5 (6)
Sepsis during the first week	5 (6)
Deceased during first week	2 (2)
Deceased during NICU stay	6 (6)
Number of samples per infant	19 (14, 23)

Values are presented as median (IQR) or *n* (%). IQR—interquartile range, NICU—neonatal intensive care unit, RDS—respiratory distress syndrome.

**Table 2:** Subgroup analysis of correlation and agreement.

Parameter	No. of Samples	R	Bias (SD)	Lower LoA, Upper LoA
Per TcCO_2_ measurements range				
All (20–115 mmHg)	1828	0.64	3.6 (9.1)	−14.3, 21.4
30–60 mmHg	1576	0.60	2.3 (6.8)	−11.1, 15.7
25–70 mmHg	1724	0.65	2.9 (7.4)	−11.8, 17.6
Per age (days) at sampling *				
Day of life 1	286	0.75	1 (6.8)	−12.3, 14.4
Day of life 1–3	887	0.71	2.0 (6.7)	−11.1, 15.1
Day of life 4+	851	0.59	3.8 (8.1)	−12.0, 19.6
Per sampling mode *				
Capillary	454	0.67	3.2 (8.1)	−12.6, 19.1
Arterial	1019	0.67	2.9 (7.4)	−11.6, 17.6
Venous	88	0.72	1.8 (6.2)	−10.3, 13.9
Per mode of ventilation *				
Non-invasive ventilation ^	900	0.65	3.1 (7.1)	−10.8, 17.1
Invasive ventilation	684	0.61	2.52 (8.1)	−13.6, 18.3
HFOV	243	0.6	2.28 (9.3)	−16.1, 20.6
CMV	442	0.62	2.6 (7.9)	−12.7, 18.1

* Data are presented for TcCO_2_ measurements between 25 and 70 mmHg. ^ Non-invasive ventilation includes nasal intermittent positive pressure ventilation (NIPPV), continuous positive airway pressure (CPAP), and heated humidified high-flow nasal cannula (HHHNC). CMV—Conventional mechanical ventilation; HFOV—High-frequency oscillatory ventilation; LoA—Limit of agreement.

## Data Availability

Data are available upon reasonable request from the corresponding author.
